# Chemokine CXCL1 may serve as a potential molecular target for hepatocellular carcinoma

**DOI:** 10.1002/cam4.843

**Published:** 2016-09-28

**Authors:** Ke‐qi Han, Hui Han, Xue‐qun He, Lei Wang, Xiao‐dong Guo, Xue‐ming Zhang, Jie Chen, Quan‐gang Zhu, Hua Nian, Xiao‐feng Zhai, Ma‐wei Jiang

**Affiliations:** ^1^ Department of Oncology and PharmacyShanghai Yueyang Hosptail of Integrated Traditional Chinese and Western MedicineShanghai University of Traditional Chinese MedicineShanghai200437China; ^2^ Department of Traditional Chinese MedicineChanghai Hosptail of Second Military Medical UniversityShanghai200433China; ^3^ Department of OncologyXin‐Hua Hospital Affiliated to Shanghai Jiaotong University School of MedicineShanghai200092China

**Keywords:** Chemokines, gene expression profile, hepatocellular carcinoma, PCR array, siRNA

## Abstract

The purpose of this study was to screen for changes in chemokine and chemokine‐related genes that are expressed in hepatocellular carcinoma (HCC) as potential markers of HCC progression. Total RNA was extracted from tumor and peritumor tissues from mice with HCC and analyzed using a PCR microarray comprising 98 genes. Changes in gene expression of threefold or more were screened and subsequently confirmed by immunohistochemical analyses and western blotting. Furthermore, whether chemokine knockdown by RNA interference (RNAi) could significantly suppress tumor growth in vivo was also evaluated. Finally, total serum samples were collected from HCC patients with HBV/cirrhosis (*n *=* *16) or liver cirrhosis (*n* = 16) and from healthy controls (*n* = 16). The serum mRNA and protein expression levels of CXCL1 in primary liver cancer patients were detected by qRT‐PCR and western blot analysis, respectively. Several genes were up‐regulated in tumor tissues during the progression period, including CXCL1, CXCL2, CXCL3, and IL‐1*β*, while CXCR1 expression was down‐regulated. CBRH‐7919 cells carrying CXCL1 siRNA resulted in decreased tumor growth in nude mice. The differences in serum CXCL1 mRNA and protein levels among the HCC, hepatic sclerosis (HS), and control groups were significant (*P *< 0.001). The mRNA and protein levels of CXCL1 in the HCC group were up‐regulated compared with the HS group or the control group (*P *< 0.001). Several chemokine genes were identified that might play important roles in the tumor microenvironment of HCC. These results provide new insights into human HCC and may ultimately facilitate early HCC diagnosis and lead to the discovery of innovative therapeutic approaches for HCC.

## Introduction

Hepatocellular carcinoma (HCC) is the fifth most common cancer and the third leading cause of cancer‐related mortality worldwide 1. The incidence of HCC varies considerably by geographic area, which also accounts for differences in major causative and risk factors. HCC is especially common in Asia due to the high prevalence of hepatitis B and C viruses (HBV and HCV) and the intake of dietary aflatoxin. The majority of HCC cases in China are caused by HBV infection 2, 3, 4.

Recent studies have shown that inflammation is one of the main characteristics of HCC. Inflammation is mediated in part by chemokines, which is one of the several important factors in the tumor microenvironment. The tumor microenvironment promotes tumor cell proliferation, survival, and migration and consists primarily of cancer cells, tumor‐associated fibroblasts, and chemokines 5, 6, 7, 8.

Chemokines are small soluble molecular proteins that can induce the chemotaxis of multiple cell types, including tumor cells, leukocytes, and endothelial cells. Chemokines and chemokine receptors are divided into the CXC, CC, C, and CX3C families according to the positions of their two conserved N‐terminal cysteine residues. Chemokines play a crucial role in tumor progression, angiogenesis, invasion, and metastasis, and HCC cells express a number of chemokines and chemokine receptors, including CXCL12‐CXCR4, CX3CL1‐CX3CR1, and CCL20‐CCR6 8, 9, 10. However, little is known about the complex functions and possible signaling pathways related to chemokines in HCC patients 9, 10, 11.

In this study, we evaluated chemokine and chemokine receptor expression in tumor and peritumor tissues from nude mice that were subcutaneously injected with liver cancer cells. We found that the expression of several chemokines changed over the course of tumor progression, including CXCL1. We evaluated whether knockdown of CXCL1 by RNA interference (RNAi) could suppress tumor growth in vivo. We also collected total serum samples from HCC patients with HBV/cirrhosis (*n* = 16) or liver cirrhosis (*n* = 16) and from healthy controls (*n* = 16), and measured the mRNA and protein expression of CXCL1 by qRT‐PCR and western blot analysis, respectively.

## Material and Methods

### Cell cultures

The human HCC cell line CBRH‐7919 (Chinese Academy of Science, Shanghai, China) was used in this study. The cells were cultured at 37°C in a humidified atmosphere with 5% CO_2_ in Dulbecco's modified eagle's medium (DMEM, Gibco BRL, Rockville, MD) supplemented with 10% fetal bovine serum (Life Technologies, Carlsbad, CA), 100 mg/mL penicillin G, and 50 *μ*g/mL streptomycin (Life Technologies).

### Animal model

Male Balb/c nude mice were obtained from the Laboratory Animal Service Center of the Medical College of Shanghai. All mice were maintained under specific pathogen‐free conditions and had free access to sterilized food and autoclaved water. The experimental procedures were approved by the Animal Ethics Committee of the Medical College of Shanghai.

Male Balb/c nude mice (aged 4 weeks) weighing 15–18 g were injected subcutaneously with 0.1 mL of a CBRH‐7919 cell suspension (1 × 10^7^ cells) using a 21‐gauge needle. Xenograft tumor transplantations were performed using 6‐week‐old athymic male Balb/c nude mice weighing 18–22 g. The mice were killed after 2 weeks, and their tumors were excised and measured. A portion of the tumor tissue was fixed in 10% formalin for subsequent histological examination; the remaining tissue was snap frozen in liquid nitrogen and stored at −70°C for the molecular studies.

### Collection and preparation of serum samples

With patient consent and approval by the ethical committee, serum samples were obtained from the Department of Oncology, Gastroenterology and Physical examination center of Shanghai Yueyang Hospital of Integrated Traditional Chinese and Western Medicine, Shanghai University of Traditional Chinese Medicine, China. The three groups contained 16 patients with hepatitis B‐related HCC (the presence of HBsAg, HBeAg, and HBV DNA; mean age of 68 years), 16 patients with liver cirrhosis (mean age of 67 years), and 16 healthy donors (mean age of 68 years). All serum samples were separated and divided into 10‐mL aliquots and stored at −80°C until analysis.

### Microarray

HCC tissue samples were dissolved in 1 mL of TRIzol reagent (Invitrogen, Carlsbad, CA) and homogenized. The sample volumes did not exceed 10% of the TRIzol reagent volume used for homogenization. The homogenized samples were incubated for 5 min at 15–30°C to completely dissociate the nucleoprotein complexes. The samples were then mixed with 0.2 mL of chloroform, incubated for 2–3 min at 15–30°C, and centrifuged at 12,000*g* for 15 min at 4°C. The RNA in the aqueous phase was then transferred to a new RNA‐free tube and mixed with 0.5 mL of isopropyl alcohol. The samples were incubated for another 10 min at 15–30°C and then centrifuged at 12,000*g* for 10 min at 4°C. The resulting supernatant was removed, and the RNA pellet was washed with 75% ethanol, redissolved in RNase‐free water, and stored at −70°C. All RNA samples used in this study were treated with MinElute^™^ (Qiagen, Austin, TX) to remove residual DNA. IL‐4, IL‐6, and CXCL1 RNA expression were analyzed using an RNA chip and a Model 2100 bioanalyzer (Agilent Technologies, Santa Clara, CA) using a 260/280 ratio of 1.8–2.0.

### Histologic evaluation

Formalin‐fixed tumor tissues were embedded in paraffin and cut into 4‐*μ*m sections. The sections were then stained with hematoxylin and eosin (HE) as previously described [12].

### Immunohistochemistry

Paraffin blocks were cut into 6‐*μ*m sections and placed on Superfrost Plus Microslides. The sections were deparaffinized using citric acid buffer (pH 6.0, 15 min at 95°C). The slides were treated with 3% hydrogen peroxide in methanol to block endogenous peroxidase activity and then incubated with 1% bovine serum albumin (BSA) for 20 min. Next, the slides were incubated overnight at 4°C with anti‐human primary antibodies against the following chemokines: CXCL1 (Ab86436; goat polyclonal, 1/250 diluted in 1% BSA), CXCL2 (Ab25130; goat polyclonal, 1/250 diluted in 1% BSA), CXCL3 (Ab10064; goat polyclonal, 1/250 diluted in 1% BSA), CXCR1 (Ab60254; goat polyclonal, 1/250 diluted in 1% BSA). All primary antibodies were obtained from Santa Cruz Biotechnology (Santa Cruz, CA). The slides were then incubated with 2 *μ*g/mL biotinylated anti‐goat IgG secondary antibody (Vector Laboratories, Burlingame, CA) for 40 min at room temperature. The sections were stained using the Standard Ultra‐Sensitive ABC Peroxidase Staining Kit (Pierce/Thermo Fisher Scientific, San Jose, CA) and 3, 3′‐ diaminobenzidine (Vector Laboratories), counterstained with hematoxylin, dehydrated, and then mounted with a coverslip.

### Quantitative real‐time PCR (qRT‐PCR)

Total RNA was isolated with TRIzol (Invitrogen) according to the manufacturers' protocol. The quality of the RNA was assessed using an Agilent 2100 bioanalyzer (Agilent Technologies), and the concentration was measured using a NanoDrop 2000 spectrophotometer (Thermo Fisher Scientific Inc., Wilmington, DE). The RNA was converted to cDNA using the RevertAid First Strand cDNA Synthesis Kit (Thermo Fisher Scientific, Inc., MA). The level of CXCL1 mRNA expression was evaluated by qRT‐PCR. The following primers were used for qRT‐PCR: CXCL1, 5‐'TAGAAGGTGTTGAGCGGGAAG‐3′ (sense) and 5′‐TGAGACGAGAAGGAGCATTGG‐3′ (antisense); GAPDH, 5′‐GTCGGTGTGAACGGATTTG‐3′ (sense) and 5′‐TCCCATTCTCAGCCTTGAC‐3′ (antisense). qRT‐PCR was performed using the DyNAmo ColorFlash SYBR Green qPCR Kit with an ABI 7300 system (Applied Biosystems, Foster City, CA).

### RNA interference

The siRNAs against CXCL1 were designed and ordered from Shanghai GenePharma Co., Ltd. CBRH‐7919 cells were transfected with the siRNAs using Lipofectamine RNAi Max (Invitrogen) according to the manufacturer's protocol. The cells were incubated for 48 h, and the knockdown efficiency was determined by both qRT‐PCR and western blot analysis. The siRNA with the sequence 5′‐GTCTCAGGACAGAGAAGTT‐3′ showed the most efficient knockdown of CXCL1 and was used in the present analyses.

### Western blotting

HCC cell extracts from tumor and peritumor tissues were analyzed using antibodies against CXCL1 (Ab86436; Abcam Inc., Cambridge, MA, USA), CXCL2 (Ab25130; Abcam Inc., Cambridge, MA, USA), CXCL3 (Ab10064; Abcam Inc., Cambridge, MA, USA), and their chemokine receptor CXCR1 (Ab60254; Abcam Inc., Cambridge, MA, USA). Equal amounts of protein were electrophoresed in sodium dodecyl sulfate‐polyacrylamide gels, and *β*‐actin was used as a loading control. Immunoreactive bands were developed on an X‐ray film using an ECL Western Blotting Analysis System (Amersham, Buckinghamshire, UK) according to the manufacturer's instructions.

### Xenografts in nude mice

Male Balb/c nude mice (4 weeks old, 15–18 g) were ordered from the Laboratory Animal Service Center of the Medical College of Shanghai. The mice were randomly assigned to the experimental or control group (*n* = 6). CBRH‐7919 cells (2 × 10^7^ cells/mouse) were injected into the left flank of the mice. The tumor sizes were measured using a caliper twice weekly. The tumor volume was calculated according to the following formula: 0.5 × length × width^2^.

### Statistical analyses

The data are summarized as the mean ± SEM (standard error of the mean). All statistical calculations were performed using either Student's *t‐*tests or Wilcoxon's rank‐sum tests in MedCalc (Mariakerke, Belgium). *P *< 0.05 was considered statistically significant.

## Results

### Establishment of an in vivo HCC mouse model with CBRH‐7919 cells

We subcutaneously injected CBRH‐7919 cells into nude mice to establish an in vivo mouse model of HCC. The mice were evaluated for tumor formation every 3 or 4 days. Local tumors were first observed in the subcutaneous abdominal regions of the mice at 7 days after injection.

**Figure 1:**
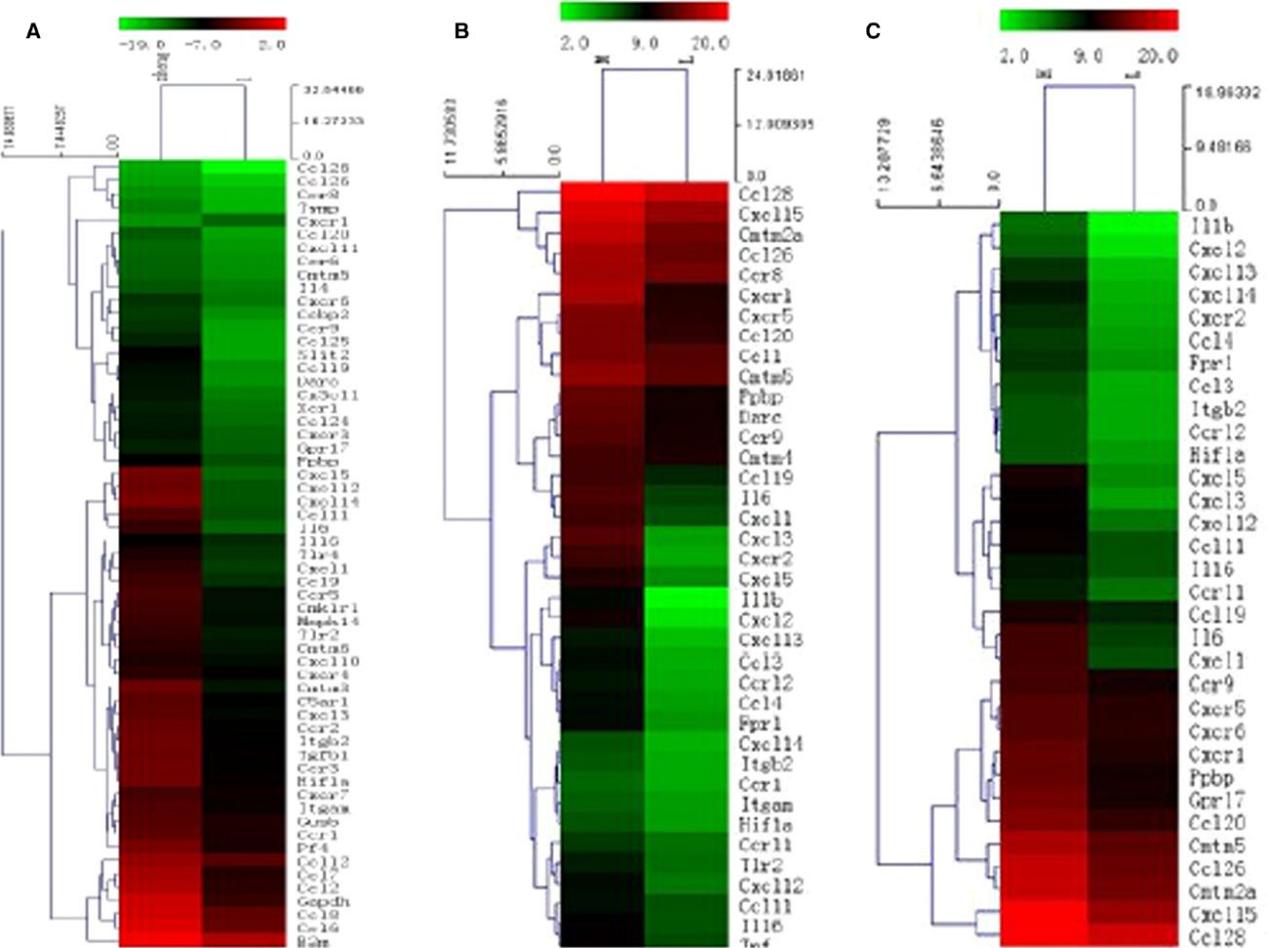
Hierarchical clustering analysis and line plots of genes with altered expression in tumor tissues compared with peritumor tissues at 1 week (A), 2 weeks (B), and 3 weeks (C). Expression profiles of chemokine‐associated genes were obtained by PCR microarray. The relative changes in gene expression are represented by colors, with red representing up‐regulation and green representing down‐regulation. Two‐dimensional hierarchical clusters were prepared with GeneSpring 6.1 and Gene Tree View using these gene expression profiles.

Comparative analyses of the gene profiles of chemokines and chemokine receptors between tumor and peritumor tissues at 1, 2, and 3 weeks, are shown in Figure 1.

### Expression levels of chemokine‐related genes in HCC

**Figure 2:**
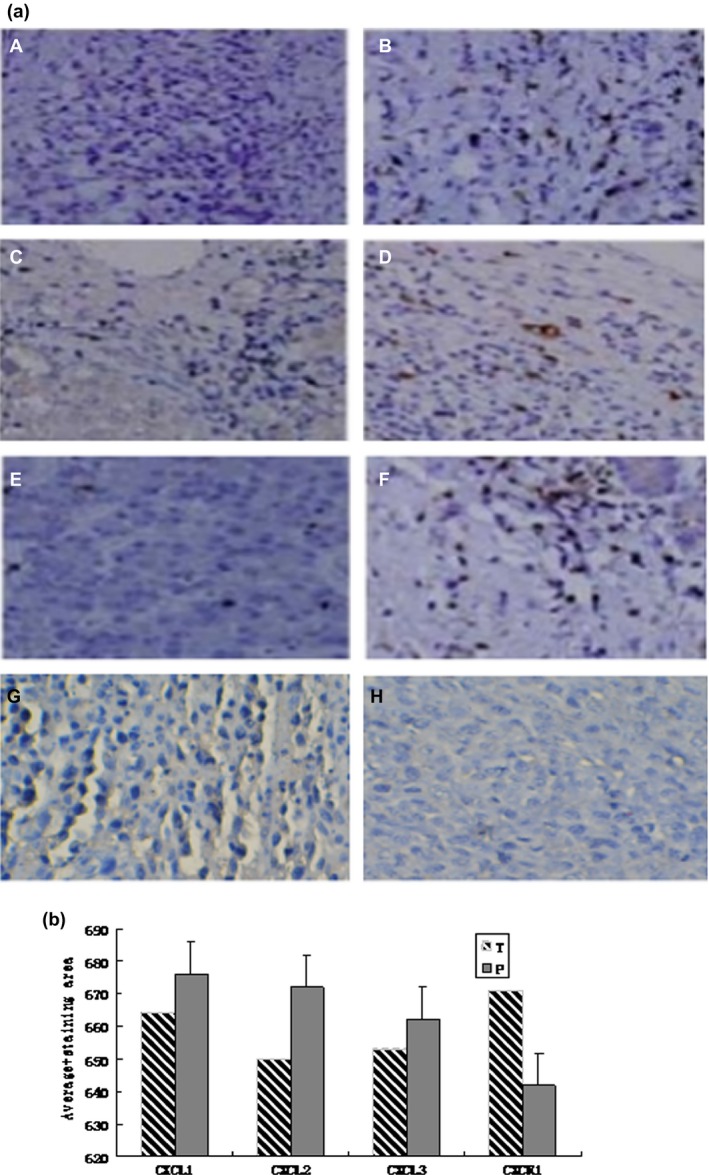
CXCL1, CXCL2, CXCL3, and CXCR1 expression in carcinoma cells xenografted into nude mice at 2 weeks. CXCL1, CXCL2, CXCL3, and CXCR1 expression were examined by immunohistochemistry in tumor and peritumor tissues. Intense CXCL1 staining was observed in tumor tissue (B) compared with peritumor tissue (A). Similar patterns were observed for CXCL2 (D, C), CXCL3 (F, E), and CXCR1 (H, G). All images were captured at 200× using a Nikon microscope equipped with a digital camera.

The expression levels of chemokine‐related genes during HCC progression were evaluated using tumor and peritumor tissues that were freshly isolated from nude mice at 1, 2, and 3 weeks. A chemokine PCR microarray comprising 98 genes was used to analyze the up‐ or down‐regulation of these genes in tumor versus peritumor tissues. We identified several genes that were up‐regulated and one gene that was down‐regulated at least threefold (Fig. 2) in the tumor tissue. Specifically, the chemokine‐related genes, CXCL1, CXCL2, CXCL3, and IL‐1*β*, were up‐regulated, while CXCR1 was down‐regulated in tumor tissue during the progression of HCC (Tables 1, 2, 3).

**Table 1:** Up‐ and down‐regulated chemokine‐related genes in tumor tissues compared with peritumoral tissues in 3 weeks

Accession no.	Gene name	Gene symbol	Fold
A01	C5ar1	Complement component 5a receptor 1	8.86
A02	Ccbp2	Chemokine‐binding protein 2	15.39
A04	Ccl11	Chemokine (C‐C motif) ligand 11	97.95
A05	Ccl12	Chemokine (C‐C motif) ligand 12	4.75
A07	Ccl19	Chemokine (C‐C motif) ligand 19	71.66
A08	Ccl2	Chemokine (C‐C motif) ligand 2	25.81
A11	Ccl24	Chemokine (C‐C motif) ligand 24	17.46
B06	Ccl6	Chemokine (C‐C motif) ligand 6	20.28
B07	Ccl7	Chemokine (C‐C motif) ligand 7	22.47
B08	Ccl8	Chemokine (C‐C motif) ligand 8	16.51
B09	Ccl9	Chemokine (C‐C motif) ligand 9	20.72
B10	Ccr1	Chemokine (C‐C motif) receptor 1	5.20
C01	Ccr2	Chemokine (C‐C motif) receptor 2	14.64
C02	Ccr3	Chemokine (C‐C motif) receptor 3	14.14
C04	Ccr5	Chemokine (C‐C motif) receptor 5	9.77
C05	Ccr6	Chemokine (C‐C motif) receptor 6	4.40
C11	Cmklr1	Chemokine‐like receptor 1	8.96
D01	Cmtm3	CKLF‐like MARVEL transmembrane domain containing 3	28.58
D03	Cmtm5	CKLF‐like MARVEL transmembrane domain containing 5	4.14
D04	Cmtm6	CKLF‐like MARVEL transmembrane domain containing 6	5.27
D05	Cx3 cl1	Chemokine (C‐X3‐C motif) ligand 1	32.58
D07	Cxcl1	Chemokine (C‐X‐C motif) ligand 1	23.49
D08	Cxcl10	Chemokine (C‐X‐C motif) ligand 10	3.15
D09	Cxcl11	Chemokine (C‐X‐C motif) ligand 11	4.84
D10	Cxcl12	Chemokine (C‐X‐C motif) ligand 12	246.94
D12	Cxcl14	Chemokine (C‐X‐C motif) ligand 14	323.14
E04	Cxcl3	Chemokine (C‐X‐C motif) ligand 3	12.76
E05	Cxcl2	Chemokine (C‐X‐C motif) ligand 2	458.57
E07	Cxcr1	Chemokine (C‐X‐C motif) receptor 1	−4.18
E09	Cxcr3	Chemokine (C‐X‐C motif) receptor 3	12.42
E10	Cxcr4	Chemokine (C‐X‐C motif) receptor 4	3.16
E12	Cxcr6	Chemokine (C‐X‐C motif) receptor 6	9.26
F01	Cxcr7	Chemokine (C‐X‐C motif) receptor 7	3.60
F02	Darc	Duffy blood group, chemokine receptor	68.40
F04	Gpr17	G protein‐coupled receptor 17	7.55
F05	Hif1a	Hypoxia‐inducible factor 1, alpha subunit	12.83
F07	Il16	Interleukin 16	3.96
F08	IL‐1beta	Interleukin 1 beta	10.67
F10	Il6	Interleukin 6	87.67
F11	Itgam	Integrin alpha M	5.51
F12	Itgb2	Integrin beta 2	15.70
G02	Mapk14	Mitogen‐activated protein kinase 14	6.74
G03	Pf4	Platelet factor 4	9.21
G04	Ppbp	Proplatelet basic protein	14.62
G06	Tgfb1	Transforming growth factor, beta 1	12.46
G07	Tlr2	Toll‐like receptor 2	8.67
G08	Tlr4	Toll‐like receptor 4	10.41
G10	Tymp	Thymidine phosphorylase	6.22
G12	Xcr1	Chemokine (C motif) receptor 1	17.78
H02	Actb	Beta‐2 microglobulin	6.14
H03	B2 m	Glyceraldehyde‐3‐phosphate dehydrogenase	41.27
H04	Gapdh	Glucuronidase, beta	3.63

**Table 2:** Up‐ and down‐regulated chemokine‐related genes in tumor tissues compared with peritumoral tissues in 2 weeks

Accession no.	Gene name	Gene symbol	Fold
A04	Scya11	Chemokine (C‐C motif) ligand 11	4.35
A06	Abcd‐2	Chemokine (C‐C motif) ligand 17	4.10
A07	CKb11	Chemokine (C‐C motif) ligand 19	4.04
B08	AI323594	Chemokine (C‐C motif) ligand 2	6.46
C05	CC‐CKR‐6	Chemokine (C‐C motif) receptor 6	3.88
D05	AB030188	Chemokine (C‐X3‐C motif) ligand 1	4.81
D10	AI174028	Chemokine (C‐X‐C motif) ligand 12	5.12
D12	1110031L23Rik	Chemokine (C‐X‐C motif) ligand 14	18.70
G12	Ccxcr1	Chemokine (C motif) receptor 1	4.76
B03	AI323804	Chemokine (C‐C motif) ligand 3	−6.32
B04	AT744.1	Chemokine (C‐C motif) ligand 4	−3.61
C10	1810047I05Rik	Chemokine (C‐C motif) receptor‐like 2	−8.32
E03	Gro2	Chemokine (C‐X‐C motif) ligand 2	−38.56
E08	CD128	Chemokine (C‐X‐C motif) receptor 2	−45.38
E11	CXC‐R5	Chemokine (C‐X‐C motif) receptor 5	−4.55
F03	FPR	Formyl peptide receptor 1	−3.31
F08	IL‐1beta	Interleukin 1 beta	3.88
G04	Gur	Glucuronidase	−5.84
D07	Cxcl1	Chemokine (C‐X‐C motif) ligand 1	14.59
E04	Cxcl3	Chemokine (C‐X‐C motif) ligand 3	5.366
E05	Cxcl2	Chemokine (C‐X‐C motif) ligand 2	158.00

**Table 3:** Up‐ and down‐regulated chemokine‐related genes in tumor tissues compared with peritumoral tissues in 1 week

Accession no.	Gene name	Gene symbol	Fold
A01	C5aR	Complement component 5a receptor 1	3.61
A02	AI464239	Chemokine‐binding protein 2	4.14
A04	Scya11	Chemokine (C‐C motif) ligand 11	15.67
A06	Abcd‐2	Chemokine (C‐C motif) ligand 17	5.71
A07	CKb11	Chemokine (C‐C motif) ligand 19	54.42
A10	ABCD‐1	Chemokine (C‐C motif) ligand 22	3.24
B03	AI323804	Chemokine (C‐C motif) ligand 3	3.50
B04	AT744.1	Chemokine (C‐C motif) ligand 4	4.60
B10	Cmkbr1	Chemokine (C‐C motif) receptor 1	3.06
C01	Cc‐ckr‐2	Chemokine (C‐C motif) receptor 2	4.38
C02	CC‐CKR3	Chemokine (C‐C motif) receptor 3	4.12
C04	AM4‐7	Chemokine (C‐C motif) receptor 5	3.58
C05	CC‐CKR‐6	Chemokine (C‐C motif) receptor 6	3.83
C06	CD197	Chemokine (C‐C motif) receptor 7	3.22
D01	9430096L06Rik	CKLF‐like MARVEL transmembrane domain containing 3	3.73
D07	Cxcl1	Chemokine (C‐X‐C motif) ligand 1	14.59
D05	AB030188	Chemokine (C‐X3‐C motif) ligand 1	4.58
D10	Scyb12	Chemokine (C‐X‐C motif) ligand 12	39.42
D11	4631412M08Rik	Chemokine (C‐X‐C motif) ligand 13	24.15
E02	0910001K24Rik	Chemokine (C‐X‐C motif) ligand 16	3.47
E04	Cxcl3	Chemokine (C‐X‐C motif) ligand 3	9.56
E05	Cxcl2	Chemokine (C‐X‐C motif) ligand 2	318.07
E08	CD128	Chemokine (C‐X‐C motif) receptor 2	3.45
F02	FPR	Chemokine receptor	16.50
F03	AI853548	Formyl peptide receptor 1	4.36
F05	AA959795	Hypoxia‐inducible factor 1	3.82
F06	IFN‐g	Interferon gamma	3.30
F07	KIAA4048	Interleukin 16	4.62
F08	IL‐1beta	Interleukin 1 beta	5.67
F09	Il‐4	Interleukin 4	5.18
F10	Il‐6	Interleukin 6	19.78
F11	CD11b	Integrin alpha M	3.59
F12	2E6	Integrin beta 2	6.60
G09	TNF‐alpha	Tumor necrosis factor	3.19
G12	Ccxcr1	Chemokine (C motif) receptor 1	10.86
H03	Gapd	Glyceraldehyde‐3‐phosphate dehydrogenase	3.99

### Immunohistochemical and western blot analyses

**Figure 3:**
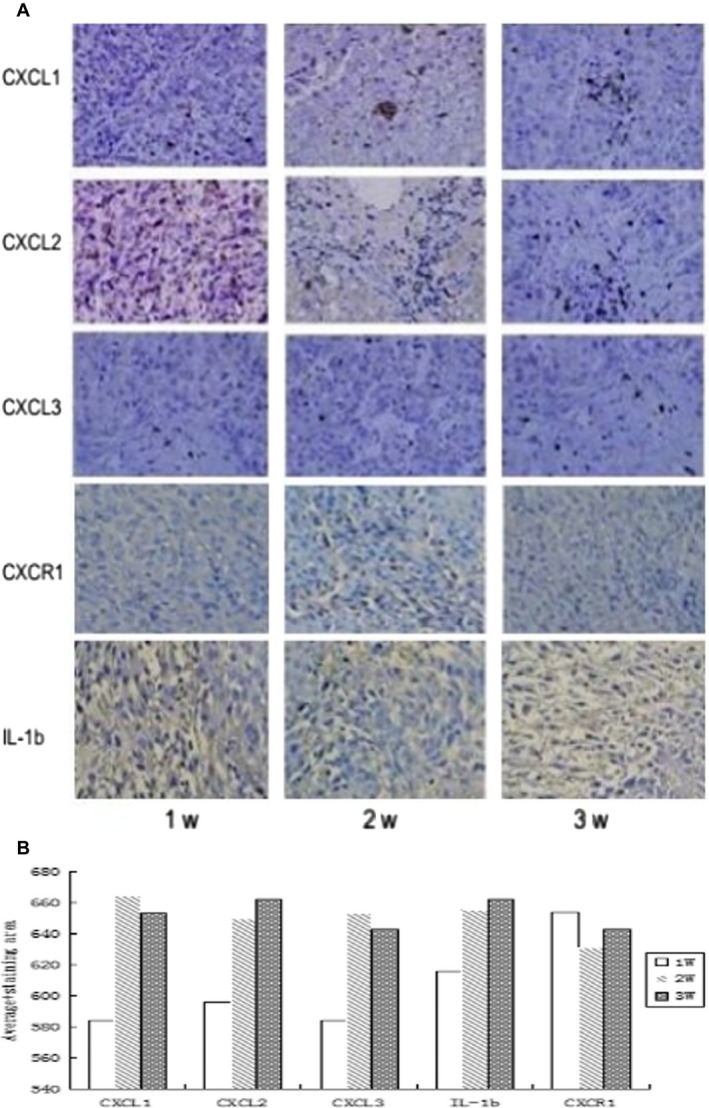
CXCL1, CXCL2, CXCL3, CXCR1, and IL‐1*β* expression in carcinoma cells xenografted into nude mice at 1, 2, and 3 weeks, respectively.

**Figure 4:**
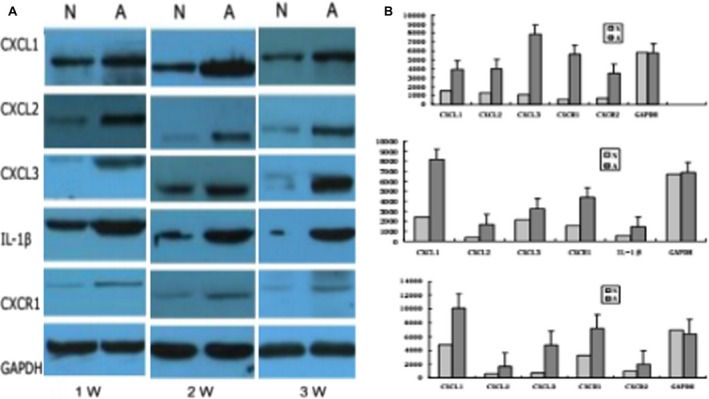
Western blot analysis of tumor and peritumor tissues from the CBRH‐7919 cell line demonstrated the up‐regulation of CXCL1, CXCL2, CXCL3, and XCR1 and down‐regulation of CXCR1 in tumor tissues at 1, 2, and 3 weeks. T: Tumor tissue, A: Peritumor tissue.

We analyzed the expression of the selected genes using immunohistochemistry (Fig. 3) and western blot analysis (Fig. 4) to confirm the changes observed by PCR microarray. The results were consistent with the microarray analysis results. CXCL1, CXCL2, and CXCL3 expression was higher and CXCR1 expression was lower in tumor tissues during HCC progression compared with the controls.

### Effect of CXCL1 knockdown on tumor growth in a xenograft model

**Figure 5:**
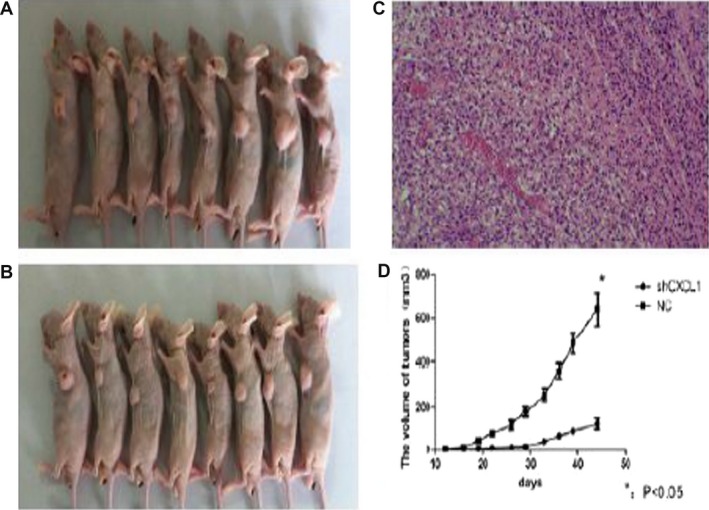
Targeted silencing of CXCL1 by siRNA. A CBRH‐7919 xenograft in nude mice. (A: NC group; B: siRNA group, 14 days after injection, 1 × 10^7^ cells). Tumor tissues from mice that received CXCL1 siRNA were stained with hematoxylin and eosin, 200× (C). Tumor growth curve was determined by the International Veterinary Information Service System (D). All data are expressed as means ± SD, *P < 0.05, (A: NC group; B: siRNA group).

We further investigated the effect of CXCL1 knockdown on tumor cell growth in vivo. One day after siRNA transfection, tumor cells were injected into nude mice (1 × 10^7^ cells). Tumors appeared at the site of inoculation within 14 days, and the mice were observed for 45 days. Both the tumor volume and weight were lower in the mice that received CXCL1 siRNA compared with the negative control siRNA‐treated mice (tumor volume, 603.4 ± 60.8 mm^3^ vs. 1095.1 ± 102.1 mm^3^; tumor weight, 283.5 ± 21.3 mg vs. 492.5 ± 43.1 mg, respectively). Tumor growth in the CXCL1 siRNA‐treated mice was significantly decreased compared with the mice that received the negative control siRNA, as observed by HE staining (Fig. 5). These results indicated that CXCL1 might be a molecular target for the treatment of HCC.

### Serum CXCL1 mRNA and protein levels in HCC

**Figure 6:**
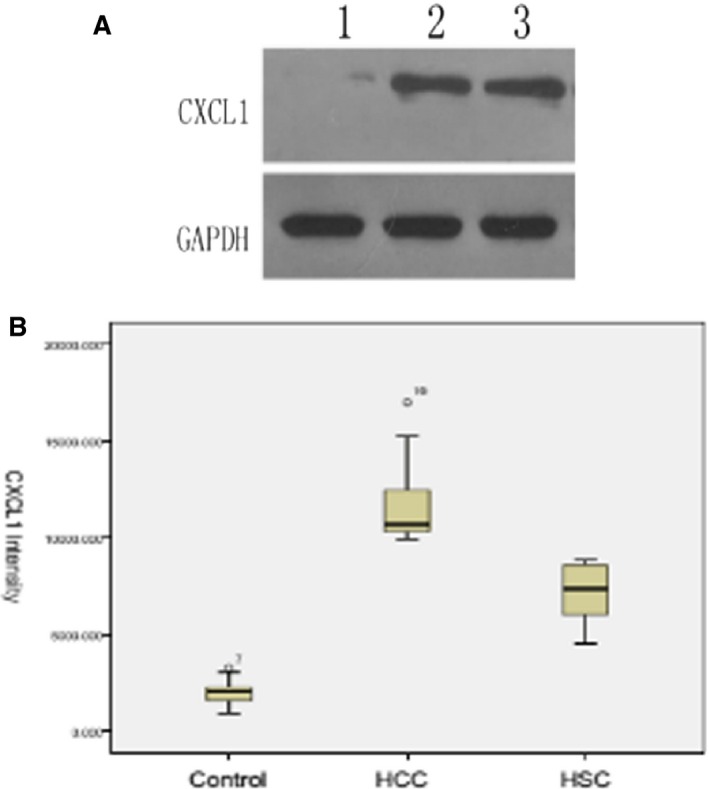
Western blot analysis of CXCL1 expression in the hepatocellular carcinoma (HCC) group (3), control group (1), and HS group (2), All data are expressed as means ± SD,* P *< 0.001, compared with the HCC group and HS group.

The results revealed a statistical difference in serum CXCL1 mRNA and protein levels among the control group, HCC group, and HS group (*P *< 0.001, Table 4). The CXCL1 mRNA and protein levels were elevated in the HCC group compared with the control group or the HS group (*P *< 0.001, Fig. 6).

**Table 4:** mRNA expression levels of CXCL1 in hepatocellular carcinoma (HCC) group, control group, and hepatic sclerosis group

	*N*	X ± S	*F*	*P*
Control	16	0.00875836 ± 0.003146918		
HCC	16	0.02627177 ± 0.005271185	53.433	<0.001
HS	16	0.01349642 ± 0.006219482		

All data are expressed as means ± SD, *P* < 0.001, as compared to HCC group and HS group.

## Discussion

Chemokines and chemokine receptors play complex roles during HCC progression. Chemokines are grouped according to their different structures and functions into four families: CC, CXC, C, and CX_3_C. CXC chemokines are the second largest family of chemokines, and increasing evidence suggests that chemokine expression is associated with tumor angiogenesis, tumor, progression, and metastasis [13, 14, 15]. CXCL1 has been shown to be a growth‐regulated oncogene with melanoma growth‐stimulating activity. Studies have shown that CXCL1 can regulate tumor epithelial–stromal interactions that facilitate tumor growth and invasion, and CXCL1 has also been associated with angiogenesis [16, 17, 18, 19]. CXCL1 is primarily regulated by growth factors/mediators, such as VEGF, TGF‐*β*, JNK, and PI3K. For example, VEGF can stimulate the release of CXCL1 in both time‐ and concentration‐dependent manner, and this phenomenon can be inhibited by VEGF receptor antagonists [20, 21, 22, 23].

Some studies have suggested that chemokines also mediate tumor metastasis. Both CXCL1 and CXCL2 are closely related to metastasis [16, 24]. CXCL1 and CXCL2 expression can be inhibited by inhibiting phosphorylated IkBa and NFkB activation. NFkB promotes the survival of premalignant epithelial cells while also stimulating the release of proinflammatory mediators. NFkB affects the expression of at least 400 genes with a variety of functions, including inflammation, invasion, and metastasis. Thus, the down‐regulation of chemokines may be a potential treatment strategy for cancer [24, 25, 26, 27].

CXCL2 and CXCL3 are up‐regulated by proinflammatory cytokines. Our previous findings suggest that CXCL1, CXCL2, and CXCL3 are up‐regulated and CXCR1 is down‐regulated in the tumors of mice with HCC. These CXC chemokines and chemokine receptors are also expressed in several solid tumors. In human colon carcinoma cell lines, CXCL1 and its receptor CXCR2 have been associated with metastatic potential and are thought to modulate cell proliferation and invasion in both an autocrine and paracrine manner. CXCL1 is also overexpressed in human bladder carcinomas, and this increased expression is associated with a higher bladder carcinoma grade and stage. CXCL1 has also been reported to be overexpressed in renal, gastric, skin, and breast cancers. However, some studies investigating CXCL1 expression in non‐small cell lung cancer have demonstrated opposite results [28, 29].

CXCR1 binds only to CXCL8. Several studies have suggested that CXCR1 is an important player in tumor progression. Neutralization of CXCR1 using small molecule antagonists affects cell proliferation and migration. Recent reports have also described CXCR1 expression in all melanoma cases, irrespective of the stage and grade, and modulation of CXCR1 expression and/or activity has been shown to regulate malignant melanoma growth, angiogenesis, and metastasis [30, 31, 32]. In addition, activation of both CXCR1 and CXCR2 increases the rate of cell proliferation in prostate cancer [33, 34]. A previous cross‐array has shown that CXCR1 is down‐regulated in HCC tissues, which is consistent with the present results. Thus, down‐regulation of CXCR1 may favor tumor progression in mouse models of HCC.

In this study, we used a PCR microarray and found that several chemokine‐related genes were up‐regulated and down‐regulated in HCC tissues. Western blotting confirmed the changes in the expression of CXCL1, CXCL2, CXCL3, IL‐1*β*, and CXCR1 in a CBRH‐7919 HCC mouse model. Further studies may increase insight into the roles of CXCL1, CXCL2, IL‐1*β*, and CXCL3 in a protumor microenvironment, which may contribute to HCC development and progression.

RNAi is a posttranscriptional gene silencing mechanism that is now widely used for the analysis and therapeutic potential of genes. RNAi has emerged as a powerful tool to induce loss of function phenotypes via posttranscriptional silencing of gene expression. A lentivirus is a retrovirus, and lentiviral vectors can efficiently deliver si/shRNA expression cassettes into various cells with sustained expression and potent functionality of the encoded siRNAs [35, 36, 37].The introduction of an siRNA targeting CXCL1 into CBRH‐7919 resulted in efficient and specific inhibition of CXCL1 expression, as demonstrated by western blotting. The results showed that gene silencing of CXCL1 inhibited the growth of CBRH‐7919 tumors in vivo (*P *< 0.01). Our findings also confirmed that the mRNA and protein levels of CXCL1 were overexpressed in patients with HCC. In conclusion, these findings support the hypothesis that CXCL1 plays an important role in protecting CBRH‐7919 cells from cell death, thus verifying the potential of CXCL1 as a target for future therapeutic interventions.

## Conflict of Interest

None declared.
